# Smartphone-Based Physical Performance and Multidimensional Determinants of Self-Reported Knee Pain in Community-Dwelling Older Adults: Cross-Sectional Machine Learning and Network Analysis Study

**DOI:** 10.2196/92466

**Published:** 2026-08-03

**Authors:** Ui-jae Hwang, Peng Xia, Tianxiang Fan, Arnold YL Wong, Oh-yun Kwon, Siu-ngor Fu

**Affiliations:** 1 Department of Rehabilitation Sciences, Hong Kong Polytechnic University, 11 Yuk Choi Road, Hung Hom, Kowloon, Hong Kong, China (Hong Kong), (+852) 60580339; 2 Department of Physical Therapy, Yonsei University, Wonju-si, Republic of Korea

**Keywords:** machine learning, network analysis, knee pain, aging, digital health, geriatric assessment

## Abstract

**Background:**

Knee pain affects 22.9% of individuals aged 40 years and over globally and is associated with body function, activity, environmental, and personal factors described in the *International Classification of Functioning, Disability and Health* (ICF) model. Most prior studies examined isolated risk factors using conventional regression.

**Objective:**

This study aimed to examine self-reported knee pain in community-dwelling older adults by combining machine learning and partial-correlation network analysis with smartphone-based physical performance measurement.

**Methods:**

This cross-sectional study included 852 adults aged 60 years and older. Baseline assessment of 38 variables across 7 ICF-aligned domains was reduced to 21 predictors using prespecified, rule-based criteria (events per variable=14.95). Walking speed, sit-to-stand, gait knee flexion, and gait asymmetry were measured using a validated vision-based smartphone app. Missing data were addressed by multiple imputation (m=5) with Rubin rules. Six algorithms (logistic regression with elastic-net penalty as baseline, k-nearest neighbors, random forest, Extreme Gradient Boosting, Light Gradient Boosting Machine, and support vector machine) were trained using nested 5×5 cross-validation, and the best model was interpreted using Shapley Additive Explanations and partial dependence plots. Partial-correlation network analysis examined the 10 top-ranked features and the knee-pain node, with bootstrap stability assessment and a sensitivity analysis excluding EuroQol 5-Dimension (EQ-5D) Pain/Discomfort.

**Results:**

Knee pain prevalence was 36.9% (314/852). Pooled area under the receiver operating characteristic curve values ranged from 0.692 to 0.723, with random forest highest (area under the receiver operating characteristic curve=0.723, 95% CI 0.689‐0.757). The top Shapley Additive Explanations features were EQ-5D Pain/Discomfort, EQ-5D Utility, and house estate. In the 11-node network, only EQ-5D Pain/Discomfort (partial correlation=0.260) and house estate (0.158) had direct edges with knee pain. Sit-to-stand showed the highest strength centrality (0.679) without a direct edge, acting as a hub linking body function, body structure, mental health, and activity variables. Shapley Additive Explanations importance and network strength were weakly correlated (ρ=0.018). The sensitivity analysis preserved the pattern (best area under the receiver operating characteristic curve=0.724).

**Conclusions:**

Knee pain in older adults was associated with variables spanning 7 ICF domains, with EQ-5D Pain/Discomfort and housing environment (lower-income rental estate residence) as direct correlates and sit-to-stand as a network hub. Combining machine learning and partial-correlation network analysis may inform multidisciplinary biopsychosocial assessment, pending confirmation in prospective and externally validated studies.

## Introduction

Self-reported knee pain is one of the most common musculoskeletal complaints among community-dwelling older adults and a leading contributor to disability, reduced physical activity, and impaired quality of life [1,2]. The Global Burden of Disease 2021 study estimated that 595 million people worldwide were living with osteoarthritis in 2020, corresponding to 7.6% of the global population, with knee being the most common site; compared with 2020, the number of cases of knee osteoarthritis is projected to increase by 74.9% by 2050, alongside continued increases in population aging and obesity prevalence [3,4]. Identifying which factors are associated with self-reported knee pain in community settings, before structural disease and clinical referral, is therefore a continuing research priority.

Knee pain in older adults is conceptualized as multidimensional. The *International Classification of Functioning, Disability and Health* (*ICF*) framework, built on the biopsychosocial model, organizes health into interacting domains spanning body functions and structures, activities and participation, and environmental and personal factors [5-7]. In knee osteoarthritis populations, physical performance, psychological status, sleep, and environmental context each carry independent associations with pain experience and functional limitation [8-10]. The *ICF* specifies that health outcomes such as knee pain arise from a structure in which some variables show direct associations with the outcome and others act through intermediate variables. Although this direct-versus-indirect distinction is part of the framework, prior studies of knee pain have not quantitatively separated the two types of association in a single analysis. As a result, it remains unclear which domains carry direct associations with knee pain in older adults and which contribute through other variables in the multidimensional structure.

Two analytical paradigms have been applied to multidimensional pain data, but they answer different questions and have typically been used in isolation. Machine learning (ML) classification with Shapley Additive Explanations (SHAP)–based explanation quantifies the marginal predictive importance of each feature, capturing potentially nonlinear contributions that traditional regression may miss [11,12]. Recent reviews of ML applications in knee osteoarthritis highlight the rapid growth of this methodology across clinical, structural, and surgical endpoints, while also noting that most studies focus on imaging-based outcomes and that few have addressed self-reported pain or nonimaging multidimensional determinants in older adults [2,3]. Partial-correlation network analysis characterizes the conditional dependency structure among variables, with centrality indices summarizing how each variable connects to the broader system after controlling for all others [13,14]. When the two paradigms are reported jointly on the same data, it becomes possible to determine whether a variable’s contribution to the model output is mirrored by a direct conditional dependency with the outcome, or whether that contribution is accounted for by other variables in the network. This joint use operationalizes the direct and indirect pathways described by the *ICF*, but applications of this combined analysis to knee pain remain limited [8,15]. Mobile health measures of physical performance, such as smartphone-based gait and sit-to-stand assessments [16], need to be integrated with questionnaire-based body function and environmental variables within a single *ICF*-aligned analysis.

The aim of this study was to characterize how variables across seven *ICF*-aligned domains, including smartphone-derived physical performance, are associated with self-reported knee pain in community-dwelling older adults, and to distinguish those that show direct conditional dependencies with knee pain from those whose contribution is accounted for by other variables in the multidimensional structure. We hypothesized that the seven *ICF* domains would show heterogeneous patterns of association with knee pain, with some domains contributing through direct conditional dependencies and others through indirect pathways involving other variables, in line with the multidimensional structure described by the framework.

## Methods

### Study Design and Participation

**Figure 1.**
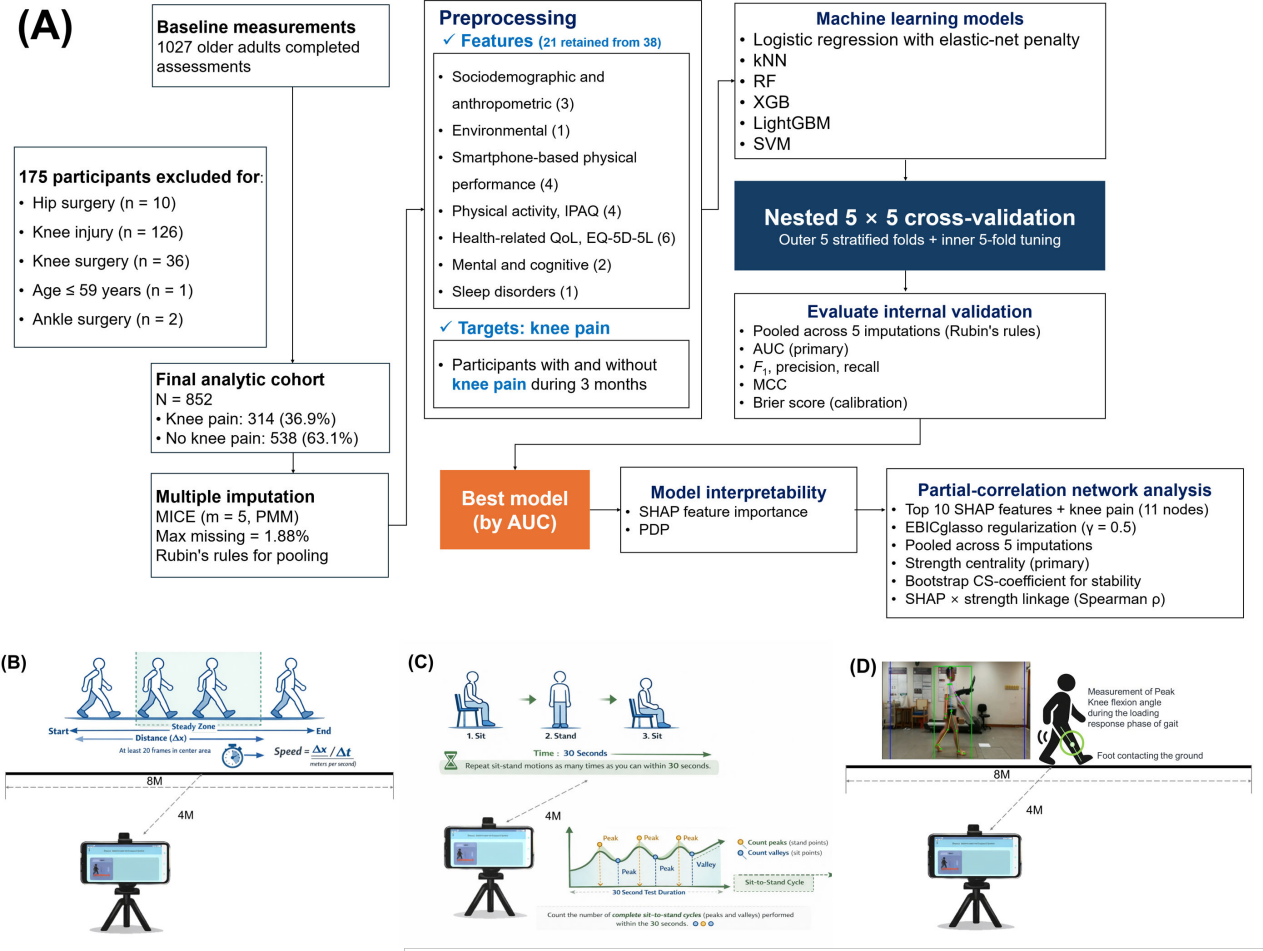
Study flowchart and analytical framework and smartphone-based physical performance assessment. (A) Study flowchart. (B) Walking speed measurement across an 8-meter walkway with smartphone positioned 4 meters perpendicular to walking path. (C) Sit-to-stand performance assessment with automated cycle counting over 30 seconds using smartphone-based motion analysis. (D) Knee flexion angle measurement during early stance phase of gait using computer vision pose estimation to detect loading response. AUC: area under the receiver operating characteristic curve; EQ-5D-5L: EuroQol 5-Dimensions 5-Levels questionnaire; IPAQ: International Physical Activity Questionnaire; kNN: k-nearest neighbors; LightGBM: Light Gradient Boosting Machine; MCC: Matthews correlation coefficient; MICE: multiple imputation by chained equations; PDP: partial dependence plots; PMM: predictive mean matching; QoL: quality of life; RF: random forest; SHAP: Shapley Additive Explanations; SVM: support vector machine; XGB: Extreme Gradient Boosting.

A total of 1027 community-dwelling Hong Kong residents aged 60 years or older were recruited for the study. Eligible participants were those living independently in housing estates, able to walk independently for at least 8 meters, and capable of providing informed consent. Exclusion criteria included (1) severe cognitive impairment preventing informed consent; (2) inability to communicate in Cantonese or read Traditional Chinese; (3) history of hip surgery, knee surgery, or ankle surgery within the past 12 months; (4) recent knee injury (within the past 6 mo); and (5) diagnosed neurological diseases including Parkinson disease, stroke, multiple sclerosis, or other conditions. Of the 1027 participants initially screened, 852 participants (83%) met all inclusion criteria and completed the comprehensive baseline assessment protocol. Study flowchart and participant characteristics are presented in Figure 1 and Table 1, respectively. Reporting follows the Transparent Reporting of a multivariable prediction model for Individual Prognosis Or Diagnosis + AI statement for prediction model studies using AI and ML [17], consistent with the *Journal of Medical Internet Research*–recommended guidelines for ML predictive models in biomedical research [18].

**Table 1.** Baseline characteristics of the analytic cohort by self-reported knee pain status.

Characteristics	Knee pain, no (n=538)	Knee pain, yes (n=314)	*P* value	Effect size
Sociodemographic and anthropometric				
Age (years), mean (SD)	76.20 (7.77)	75.53 (7.24)	.21	0.09
Gender (female), n (%)	403 (74.9)	257 (81.8)	.02	0.08
Height (m), mean (SD)	1.57 (0.08)	1.56 (0.08)	.06	0.14
Weight (kg), mean (SD)	57.00 (10.28)	58.33 (10.87)	.08	−0.13
BMI (kg/m²), mean (SD)	22.97 (3.54)	23.82 (3.84)	.001	−0.23
Environmental				
House estate, n rental (%)	336 (62.5)	258 (82.2)	<.001	0.20
Smartphone-based physical performance, mean (SD)				
Walking speed (m/s)	1.20 (0.29)	1.15 (0.26)	.02	0.17
Sit-to-stand (reps/30 s)	11.41 (3.52)	10.50 (3.16)	<.001	0.27
Gait knee flexion (degree)	20.93 (4.88)	20.41 (4.58)	.12	0.11
Gait asymmetry (%)	8.77 (7.69)	9.48 (9.10)	.25	−0.09
Physical activity (IPAQ^a^), mean (SD)				
Sitting time (min/day)	292.55 (134.27)	331.17 (138.94)	<.001	−0.28
Walking (MET^b^-min/week)	1800.28 (1519.42)	1377.88 (1090.70)	<.001	0.31
Moderate PA^c^ (MET-min/week)	1231.56 (1778.13)	1076.45 (1291.01)	.15	0.10
Vigorous PA (MET-min/week)	449.11 (1920.25)	81.92 (471.79)	<.001	0.24
Health-related quality of life (EQ-5D-5L)^d^, mean (SD)				
Mobility (1-5)	1.16 (0.47)	1.38 (0.68)	<.001	−0.39
Self-care (1-5)	1.03 (0.19)	1.04 (0.23)	.38	−0.07
Usual activities (1-5)	1.08 (0.33)	1.17 (0.47)	.003	−0.24
Pain/Discomfort (1-5)	1.41 (0.63)	1.92 (0.79)	<.001	−0.73
Anxiety/Depression (1-5)	1.19 (0.51)	1.29 (0.56)	.02	−0.17
Utility index (HK-specific)^e^	0.93 (0.11)	0.86 (0.16)	<.001	0.57
Mental and cognitive, mean (SD)				
Mental health (ReQoL^f^ total)	32.38 (5.94)	31.10 (6.13)	.006	0.21
ERA-12^g^ total	35.86 (14.68)	35.81 (15.22)	.97	0.00
Sleep, mean (SD)				
Sleep disorders (count, 0‐6)	1.08 (0.93)	1.44 (1.14)	<.001	−0.36

a IPAQ: International Physical Activity Questionnaire.

b MET: metabolic equivalent of task.

c PA: physical activity.

d EQ-5D-5L: EuroQol 5-Dimension 5-Level.

e HK: Hong Kong.

f ReQoL: Recovering Quality of Life.

g ERA-12: 12-item Expectations Regarding Aging.

### Ethical Considerations

This study was conducted in accordance with the Declaration of Helsinki and was approved by the Institutional Review Board of The Hong Kong Polytechnic University (HSEARS20220628001). All participants provided written informed consent before data collection, after the nature and possible consequences of the study were explained. Video recordings captured during the smartphone-based assessments were transmitted to institutional servers for automated pose-estimation analysis; all data were deidentified before analysis and stored in a password-protected database accessible only to the research team. No identifiable participant information is presented in this paper or its supplementary materials. As part of the wider project, participants who completed the exercise program and achieved a predefined fitness criterion at the 12-month follow-up received a health hamper valued at HKD 200 (approximately US $26).

### Knee Pain Outcome

The primary outcome was the presence of self-reported knee pain experienced within the preceding 3 months, ascertained through structured interviewer-administered questionnaires conducted by trained research assistants. Knee pain was defined as any ache, discomfort, or pain sensation experienced in one or both knees during this 3-month period [1]. Participants were classified as having knee pain (Yes) or not (No) based on this question.

### Data Collection and Measurements

#### Overview

Comprehensive baseline assessment included variables organized under seven domains aligned with the *ICF* framework: sociodemographic and anthropometric characteristics, environmental factors, smartphone-based physical performance, physical activity, health-related quality of life, mental and cognitive functioning, and sleep. All instruments used validated Traditional Chinese versions with established psychometric properties in older adult populations.

Sociodemographic and anthropometric measures comprised age, sex, and BMI (calculated from measured height and weight). Housing type (elderly housing vs rental estate) was used as an environmental indicator. The Hong Kong Housing Society operates a spectrum of estates serving different income groups, with rental housing estates serving lower-income households and senior (older) housing serving middle- to higher-income groups; in this cohort, rental estate residence therefore reflects a relatively lower socioeconomic position. Health-related quality of life was assessed using the EuroQol 5-Dimension 5-Level (EQ-5D-5L) instrument [19], confirming scores for five dimensions (Mobility, Self-care, Usual Activities, Pain/Discomfort, and Anxiety/Depression), the visual analog scale, and the Hong Kong–specific utility index. Aging expectations were assessed using the 12-item Expectations Regarding Aging questionnaire total score [20]. Mental health–related quality of life was assessed using the Recovering Quality of Life total score [21]. Sleep was assessed using the Sleep Disorders Questionnaire covering six categories (insomnia, periodic limb movement, circadian rhythm disorder, movement disorder, parasomnia, and sleep apnea); a total disorder count from 0 to 6 was used for analysis [22]. Physical activity was assessed using the International Physical Activity Questionnaire (IPAQ), from which we derived sitting time (minutes per day) and metabolic equivalent of task (MET) minutes per week for walking, moderate, and vigorous activities [23].

#### Smartphone-Based Physical Performance Assessment

Physical performance assessment was performed at community-dwelling centers using a research prototype smartphone-based digital health system that used computer vision pose estimation algorithms for mobility evaluation (Figure 1) [16]. Correlation analyses of the smartphone measurement system showed agreement for walking speed measurements (*r*=0.975) and knee flexion assessments (*r*=0.907 to 0.923), with test-retest reliability values (intraclass correlation coefficient=0.86 to 0.94) and no significant systematic bias detected following algorithm calibration [16]. The experimental setup involved positioning an Android (Google LLC) smartphone equipped with a single camera on a 0.9-meter height stand, placed 4 meters perpendicular to the midpoint of an 8-meter walkway within the dwelling centers. The smartphone was installed with a vision-based gait analysis app that uses pose estimation technology to detect and track anatomical landmarks throughout movement sequences. The app connects to a backend infrastructure featuring cloud-based processing capabilities through an Nginx load balancer (F5, Inc.) capable of handling multiple simultaneous connections. Video data is transmitted frame-by-frame in real-time to a Flask framework for pose detection [16]. Processed data, including raw video files and numerical results, are stored in a MySQL (Oracle Corporation) database with web-based researcher portals providing access to results.

For walking speed (m/s) [24,25], participants walked naturally across a standardized 8-meter walkway while being recorded using smartphone cameras positioned perpendicular to the walking path. The analysis system used human detection followed by pose estimation to track 17 key anatomical landmarks, including head, thorax, pelvis, and bilateral extremity joints with subpixel accuracy. Spatial calibration was applied by matching the median-detected human height across all frames with participant-reported height, enabling conversion from pixel coordinates to real-world measurements without external reference objects. The system automatically identified steady-state walking zones by tracking pelvis position along the trajectory, requiring consistent walking velocity within a predefined spatial range (1/5 to 4/5 of walkway length) for a minimum of 20 consecutive frames to exclude acceleration and deceleration phases. Walking speed was computed as the change in pelvis horizontal position divided by elapsed time within identified zones, with median values calculated across multiple segments and temporal smoothing applied using a 5-frame median filter.

Sit-to-stand performance was assessed through automated movement cycle counting using pose-based motion analysis over 30 seconds [26,27]. The system tracked pelvis vertical position and bilateral knee flexion angles, with hip height changes serving as the primary indicator for transitions supplemented by knee angle verification. Complete cycles were identified using peak and valley detection in hip trajectories with dynamic thresholds established based on movement range (25th to 75th percentile of hip height range) to accommodate individual differences. Each detected peak required a corresponding valley for cycle validation, counting only complete cycles within the time window from the first detected movement. Quality control included 5-frame smoothing filters and automatic switching to knee flexion angle-based analysis using predefined thresholds (110 to 160 degrees range) when hip height detection showed inconsistencies.

Bilateral knee flexion angles were measured during early stance phase using three-point angular calculations incorporating hip, knee, and ankle joint centers [28,29]. The algorithm computed angles between thigh and shank segments using arctangent-based trigonometric calculations, with early stance phase automatically identified through ankle crossing patterns and calf angle peaks. The system detected when the contralateral ankle passed the ipsilateral ankle, followed by identification of maximum calf angle relative to vertical, indicating early stance initiation. Measurements were direction-adjusted based on walking trajectory, analyzing right leg early stance for rightward walking and left leg for leftward movement. Peak knee flexion angles were identified across multiple gait cycles with median values calculated to represent typical performance while minimizing outlier influence.

### Statistical Analysis

All ML analyses were conducted using R statistical software (version 4.5.3; R Core Team). Baseline characteristics of the analytic cohort were compared between participants with and without self-reported knee pain using independent 2-tailed *t* tests (continuous variables) or *χ*^2^ tests (categorical variables).

#### Premodeling Variable Reduction

To address events-per-variable (EPV) constraints raised in the literature for prediction model development [30,31], we applied a prespecified, rule-based variable reduction prior to modeling. The 38 candidate predictor variables were reduced to 21 based on conceptual and statistical overlap (Table S1 in Multimedia Appendix 1): six Sleep Disorders Questionnaire items were collapsed into a single sleep-disorder count (0 to 6); bilateral knee flexion was averaged into one variable, with absolute bilateral asymmetry retained as a separate gait-quality measure; redundant IPAQ frequency or duration counts were dropped in favor of four MET-summary variables (sitting, walking, moderate, and vigorous); the EQ-5D visual analog scale was excluded because it overlaps with the five dimensions and the utility index; and Expectations Regarding Aging subscale scores were collapsed into the Expectations Regarding Aging-12 total. With 314 self-reported knee-pain events in the 852 analytic cohort, this supported an EPV of 14.95, exceeding the conventional threshold of 10 or greater for stable prediction model estimation [31]. Data-driven variable selection was not applied, and the rule-based reduction was conducted before any outcome-aware modeling step.

#### Multiple Imputation

Across the 21 retained variables, the maximum item-level missingness was 1.88%, with a median of 1.17% (IQR 0.47%-1.29%; Table S2 in Multimedia Appendix 1). Missing values were addressed using multiple imputation by chained equations with predictive mean matching, m=5 imputed datasets, and 30 iterations per chain. Convergence was inspected using trace plots (Figure S1 in Multimedia Appendix 1). All subsequent analyses were conducted on each of the 5 imputed datasets, and results were pooled using Rubin’s rules. The class distribution (knee pain “Yes” [314/852] 36.9%, “No” [538/852] 63.1%) constituted mild imbalance, addressed through imbalance-aware metrics (area under the receiver operating characteristic curve [AUC], *F*_1_-score, and Matthews correlation coefficient [MCC]) rather than algorithmic resampling.

#### Nested Cross-Validation and Comparison With Logistic Regression Baseline

Six classification algorithms were compared: logistic regression (LR) with elastic-net penalty (LR; clinically interpretable baseline), k-nearest neighbors, random forest (RF), Extreme Gradient Boosting (XGB), Light Gradient Boosting Machine (LightGBM), and support vector machine (SVM) with radial basis function kernel. Performance was estimated using nested 5×5 cross-validation: 5 stratified outer folds for unbiased performance estimation, with an inner 5-fold cross-validation within each outer training fold for hyperparameter grid-search tuning (Table S3 in Multimedia Appendix 1). Continuous predictors were centered and scaled within each fold. This procedure was repeated independently on each of the 5 multiply-imputed datasets, and pooled estimates with 95% CIs were obtained via Rubin rules. For each algorithm, we report pooled AUC, *F*_1_-score, precision, recall, Matthews correlation coefficient [MCC], accuracy, and Brier score with 95% CIs.

#### SHAP-Based Marginal Importance and Partial Dependence

Each variable’s marginal contribution to the model output was quantified using SHAP values computed with the fastshap package and 50 Monte Carlo permutation samples on the best-performing caret-compatible algorithm [11]. SHAP values were computed within each imputation and averaged. We report mean absolute SHAP for global ranking and beeswarm plots for per-participant distributions. To visualize the marginal model–implied relationship between each top-ranked feature and the predicted probability of knee pain, partial dependence plots (PDPs) were generated for the top 9 SHAP features using the iml package with a 30-point grid. PDPs are model-dependent visualizations that do not establish causal effects or constitute clinical thresholds; we therefore frame all PDP-derived patterns as exploratory.

#### Partial-Correlation Network Analysis With Paradigm Concordance and Pathway Decomposition

To characterize the conditional dependency structure among predictor variables and the outcome, we estimated partial-correlation networks using graphical LASSO with extended Bayesian information criterion (EBICglasso) regularization, Spearman correlations to accommodate nonnormal distributions, EBIC tuning parameter gamma of 0.5, and edge thresholding [32]. Network nodes comprised the top 10 SHAP-ranked features plus the binary knee pain outcome (n=11). This prespecified threshold was chosen to keep the node count compatible with stable EBICglasso estimation at our sample size, to focus the network on the predictors carrying the most model-relevant information, and to preserve interpretability; the sensitivity analysis confirmed a consistent network structure. One prespecified rule was applied: when EQ-5D Utility appeared in the top 10 alongside one or more individual EQ-5D dimensions, Utility was excluded (because it is a deterministic weighted combination of the dimensions) and replaced with the next-ranked SHAP feature. Networks were estimated separately on each imputation, and partial-correlation matrices were averaged. Three centrality measures were computed (strength, closeness, and betweenness); strength was prespecified as the primary measure, as closeness and betweenness are known to be unstable in cross-sectional networks of modest size. Centrality stability was assessed using case-dropping bootstrap (1000 resamples, first imputation), with the correlation-stability (CS) coefficient reported [33].

To examine paradigm concordance, we prespecified the Spearman rank correlation between SHAP importance and each centrality measure across the 10 network feature nodes (excluding the knee-pain node) as a quantitative test. We additionally decomposed each variable’s contribution into a direct pathway, operationalized as the absolute partial correlation with the knee-pain node, and an indirect pathway, operationalized as the residual SHAP magnitude after subtracting the direct edge contribution. This decomposition is descriptive, not causal: it characterizes whether a variable’s contribution to the model’s output corresponds to a direct conditional dependency with the outcome or is instead accounted for by conditional dependencies with other variables in the network.

#### Sensitivity Analysis for Outcome-Feature Conceptual Overlap

EQ-5D Pain/Discomfort is a self-rated pain item that shares conceptual content with the outcome. To examine whether model results depended on this overlap, we conducted a prespecified sensitivity analysis excluding EQ-5D Pain/Discomfort from the feature set (n=20 variables; EPV=15.7). The full pipeline was repeated unchanged, and results are reported in Table S4 in Multimedia Appendix 1.

## Results

### Baseline Characteristics

Of the 852 participants, 314 (36.9%) reported knee pain in the preceding 3 months and 538 (63.1%) did not. Baseline characteristics by knee pain status are presented in Table 1. The two groups were comparable in age (2-tailed *t*_693_=1.27; *P*=.21), with a small difference in the proportion of female participants (*χ*²_1_=5.08; *P*=.02). Participants with knee pain had higher BMI (*t*_613_=−3.22; *P*=.001), were more frequently rental estate residents (*χ*²_1_=35.57; *P*<.001), and showed slower walking speed (*t*_702_=2.41; *P*=.02) and fewer sit-to-stand repetitions (*t*_713_=3.85; *P*<.001). They reported more sitting time (*t*_635_=−3.81; *P*<.001), less walking-related (*t*_816_=4.61; *P*<.001), and vigorous physical activity (all *P*<.001), poorer health-related quality of life across multiple EQ-5D dimensions, lower mental health scores (*t*_640_=2.74; *P*=.006), and a higher count of sleep disorder categories (*t*_553_=−4.88; *P*<.001). The largest between-group difference was observed for EQ-5D Pain/Discomfort (Cohen *d*=−0.73), followed by EQ-5D Utility (Cohen *d*=0.57) and EQ-5D Mobility (Cohen *d*=−0.39).

### Model Performance

**Figure 2.**
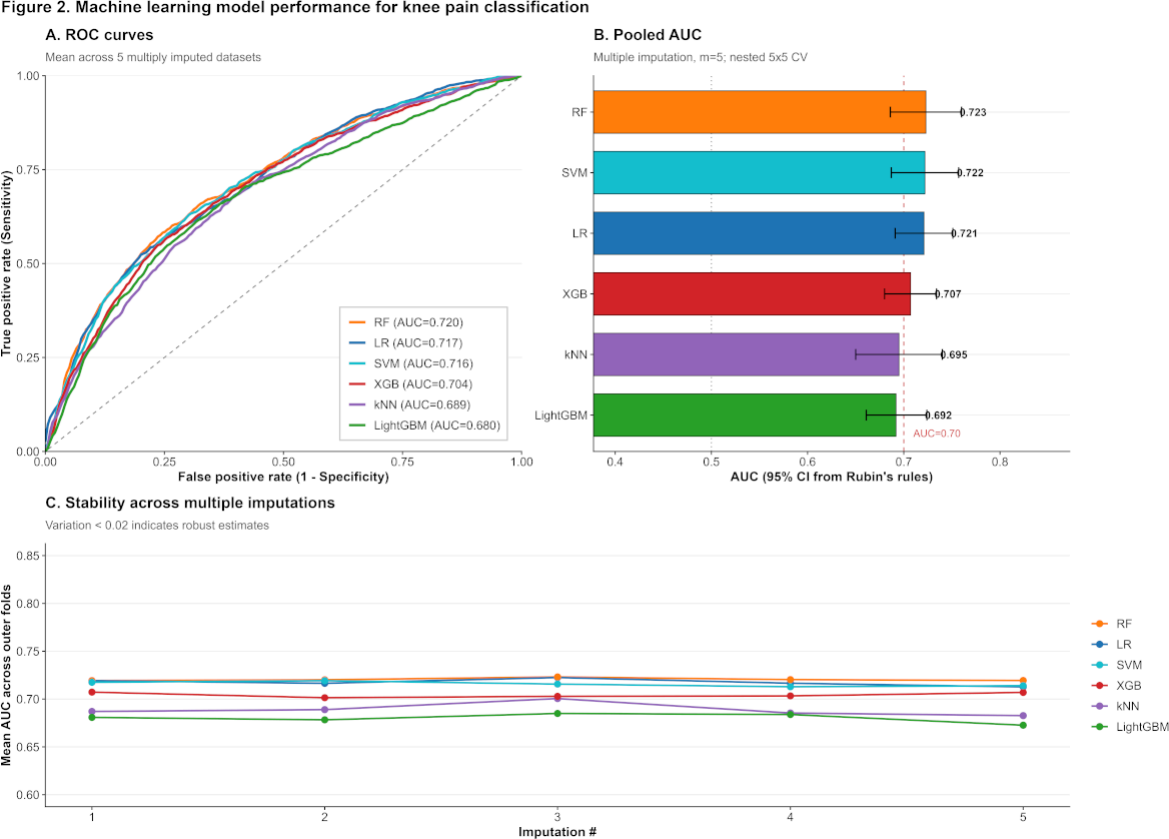
Machine learning model performance for knee pain classification. (A) Receiver operating characteristic (ROC) curves; (B) Pooled area under the ROC curve (AUC); (C) Stability across the 5 multiply-imputed datasets. AUC: area under the receiver operating characteristic curve; CV: cross-validation; ROC: receiver operating characteristic; kNN: k-nearest neighbors; LightGBM: Light Gradient Boosting Machine; LR: logistic regression with elastic-net penalty; MCC: Matthew correlation coefficient; RF: random forest; SVM: support vector machine; XGB: Extreme Gradient Boosting.

Pooled performance metrics for the six classification algorithms are presented in Table 2 and Figure 2. AUC values clustered within a narrow range from 0.692 (95% CI 0.661-0.724) for LightGBM to 0.723 (95% CI 0.686-0.760) for RF. The LR baseline showed a pooled AUC of 0.721 (95% CI 0.691-0.751), placing it within 0.002 of RF and 0.001 of SVM. *F*_1_-score, recall, and MCC were modest across all algorithms (MCC range 0.252-0.328), and LR produced the lowest Brier score (0.200), indicating the best calibration. Performance was stable across the 5 imputations, with within-imputation variation in mean AUC less than 0.02 for every algorithm (Figure 2D). The discrimination performance of all six algorithms was effectively comparable; RF was retained as the best-performing algorithm by pooled AUC for SHAP analysis.

**Table 2.** Pooled performance metrics for the six classification algorithms.

Algorithm	AUC^a^, pooled estimate (95% CI)	*F*_1_-score, pooled estimate (95% CI)	Precision, pooled estimate (95% CI)	Recall, pooled estimate (95% CI)	MCC^b^, pooled estimate (95% CI)	Brier, pooled estimate (95% CI)
RF^c^	0.723 (0.686‐0.760)	0.510 (0.473‐0.547)	0.649 (0.604‐0.694)	0.423 (0.376‐0.469)	0.325 (0.279‐0.371)	0.201 (0.191‐0.211)
SVM^d^	0.722 (0.687‐0.757)	0.523 (0.472‐0.575)	0.636 (0.584‐0.688)	0.451 (0.375‐0.527)	0.328 (0.276‐0.380)	0.202 (0.190‐0.215)
LR^e^	0.721 (0.691‐0.751)	0.466 (0.337‐0.595)	0.666 (0.597‐0.736)	0.368 (0.232‐0.505)	0.312 (0.212‐0.412)	0.200 (0.192‐0.208)
XGB^f^	0.707 (0.680‐0.734)	0.530 (0.498‐0.562)	0.598 (0.552‐0.643)	0.480 (0.429‐0.531)	0.306 (0.260‐0.351)	0.211 (0.197‐0.225)
kNN^g^	0.695 (0.650‐0.740)	0.484 (0.426‐0.542)	0.565 (0.507‐0.623)	0.427 (0.357‐0.498)	0.252 (0.179‐0.325)	0.211 (0.195‐0.227)
LightGBM^h^	0.692 (0.661‐0.724)	0.530 (0.487‐0.573)	0.574 (0.523‐0.626)	0.497 (0.434‐0.559)	0.289 (0.229‐0.350)	0.249 (0.215‐0.283)

a AUC: area under the receiver operating characteristic curve.

b MCC: Matthews correlation coefficient.

c RF: random forest.

d SVM: support vector machine.

e LR: logistic regression.

f XGB: Extreme Gradient Boosting.

g kNN: k-nearest neighbor.

h LightGBM: Light Gradient Boosting Machine

Values are pooled estimates (95% CI) across five multiply-imputed datasets, obtained via Rubin’s rules from nested 5×5 cross-validation. Algorithms are ordered by pooled AUC.

Pooled performance of six classification algorithms (LR with elastic-net penalty, k-nearest neighbors, RF, XGB, LightGBM, and SVM with radial basis function kernel) estimated using nested 5×5 cross-validation on five multiply-imputed datasets and pooled via Rubin rules.

### SHAP-Based Marginal Contribution

**Figure 3.**
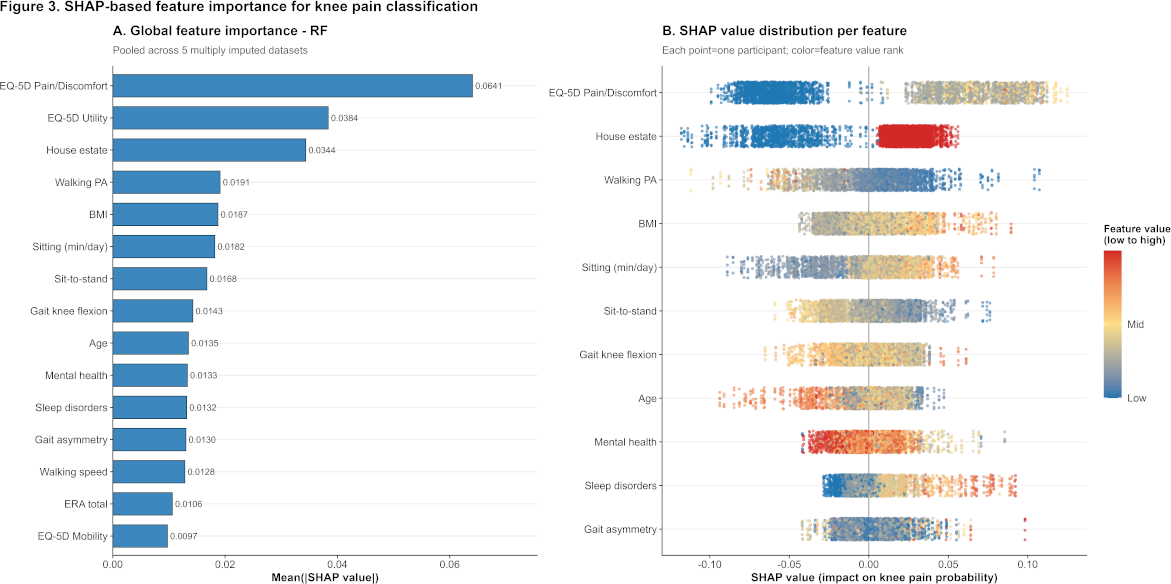
SHAP-based feature importance for knee pain classification. (A) Feature importance for mean absolute SHAP value for the top 15 features; (B) SHAP value distribution per feature (beeswarm plot). ERA: Expectations Regarding Aging; EQ-5D: EuroQol 5-Dimension; SHAP: Shapley Additive Explanations; BMI: body mass index; PA: physical activity.

Pooled SHAP-based feature importance for the RF model is presented in Figure 3, with values for all 21 variables in Table S5 in Multimedia Appendix 1. The three highest-ranked features were EQ-5D Pain/Discomfort (mean |SHAP|=0.064), EQ-5D Utility (0.038), and house estate (0.034). Subsequent features clustered at substantially lower magnitudes (|SHAP| range 0.013-0.019) and included physical activity, anthropometric, smartphone-based physical performance, and mental and sleep variables. The SHAP value distributions in Figure 3 characterized the direction of each association: higher EQ-5D Pain/Discomfort scores, lower EQ-5D Utility, rental estate residence, lower walking physical activity and sit-to-stand repetitions, higher BMI and sitting time, and higher sleep disorder counts were each associated with increased predicted probability of knee pain, consistent with the group differences observed in Table 1.

### Partial Dependence Plots

**Figure 4.**
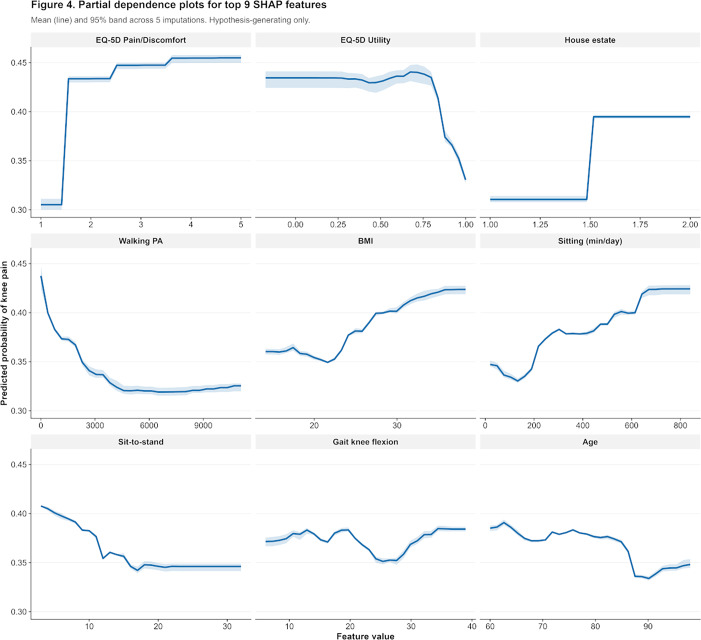
Partial dependence plots for the top 9 SHAP-ranked features. Features are arranged in descending order of SHAP importance: EQ-5D Pain/Discomfort, EQ-5D Utility, house estate, walking physical activity, body mass index, sitting time, sit-to-stand repetitions, gait knee flexion, and age. EQ-5D: EuroQol 5-Dimension 5-Level; PA: physical activity; SHAP: Shapley Additive Explanations.

PDPs for the top 9 SHAP-ranked features are shown in Figure 4. The model-implied probability of knee pain rose sharply between EQ-5D Pain/Discomfort scores of 1 and 2 (from approximately 0.31 to 0.43), reaching a plateau near 0.45. The relationship for EQ-5D Utility was relatively flat across most of the range and decreased steeply at utility values above approximately 0.85. House estate showed a step function consistent with its binary coding. For continuous features, the model-implied probability of knee pain decreased with higher walking physical activity and sit-to-stand repetitions and increased with higher BMI and sitting time. Gait knee flexion and age showed comparatively flat partial dependence functions. These patterns describe model-implied marginal relationships and are presented as exploratory; they do not establish causal effects or clinical thresholds.

### Network Structure, Paradigm Concordance, and Pathway Decomposition

**Figure 5.**
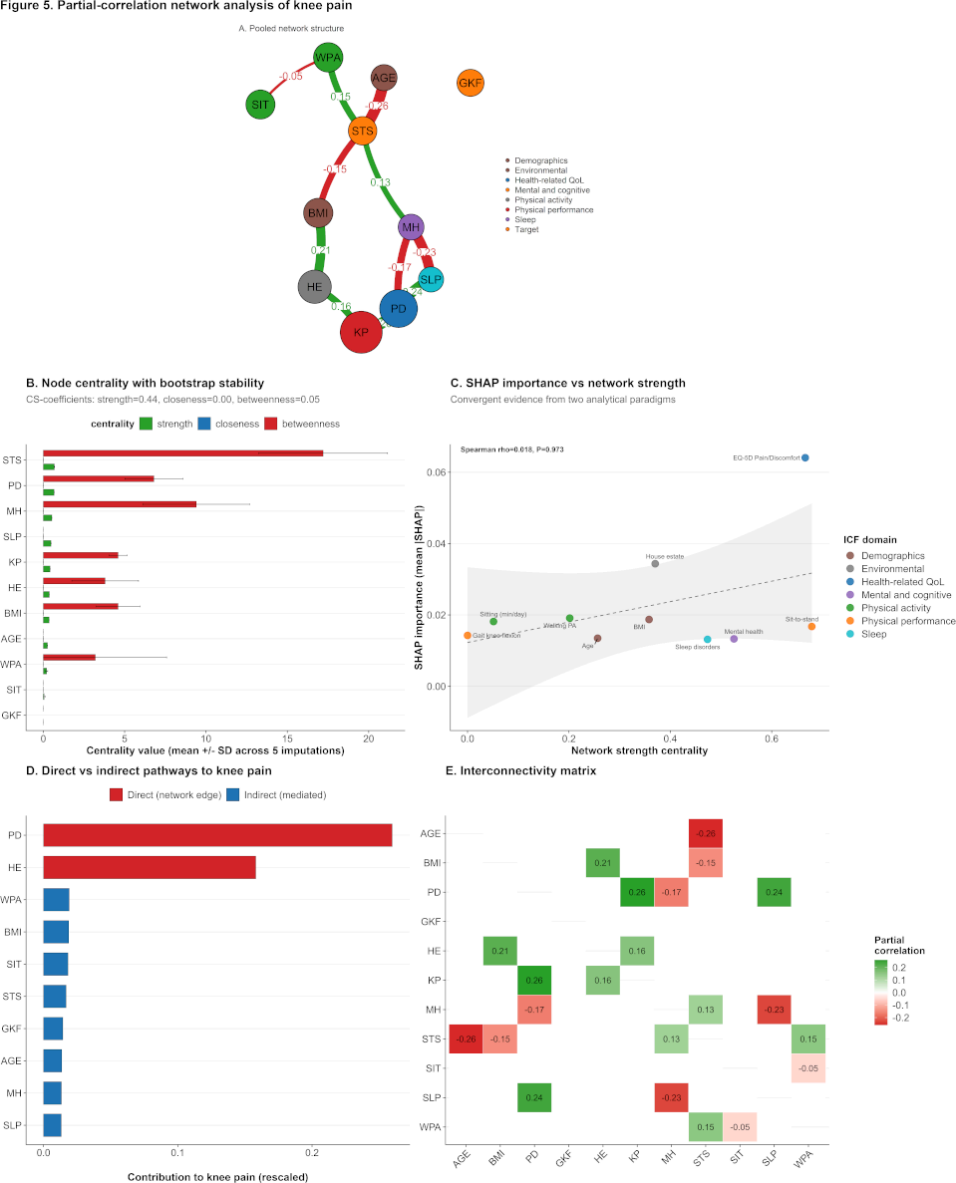
Partial-correlation network, centrality, paradigm concordance, and pathway decomposition. (A) Pooled network structure, (B) node centrality with bootstrap stability, (C) linkage between SHAP importance and network strength centrality, (D) direct versus indirect pathways to knee pain, and (E) interconnectivity matrix. CS: correlation-stability; EBICglasso: graphical LASSO with extended Bayesian information criterion regularization; GKF: gait knee flexion; HE: house estate; *ICF*: *International Classification of Functioning, Disability and Health*; KP: knee pain; LASSO: Least Absolute Shrinkage and Selection Operator; MH: mental health; ML: machine learning; PD: EQ-5D-5L Pain/Discomfort dimension; SHAP: SHapley Additive exPlanations; SIT: sitting timea; SLP: sleep disorders; STS: sit-to-stand; WPA: walking physical activity; QoL: quality of life.

The pooled partial-correlation network for the 11 nodes is shown in Figure 5A, with the full pooled partial-correlation matrix in Table S6 in Multimedia Appendix 1. After EBICglasso regularization and edge thresholding, 11 conditional dependencies survived. The highest strength centrality values were observed for sit-to-stand (0.679), EQ-5D Pain/Discomfort (0.666), and mental health (0.526), with the knee pain node ranking 5th of 11 (0.418; Figure 5). The case-dropping bootstrap CS-coefficient was 0.36 for strength (acceptable stability), and 0.00 and 0.05 for closeness and betweenness, respectively.

Two direct edges connected the knee pain node to the predictor set: a positive edge with EQ-5D Pain/Discomfort (partial correlation=0.260) and a positive edge with house estate (0.158). The remaining nine network edges represented conditional dependencies among the predictors after controlling for all others. The prespecified Spearman rank correlation between SHAP importance and network strength centrality across the 10 feature nodes was *ρ*=0.018 (*P*=.97), indicating no monotonic association between the two paradigms (Figure 5).

The decomposition of each variable’s contribution into direct and indirect pathways (Figure 5D) showed a clear binary pattern. EQ-5D Pain/Discomfort and house estate carried their contributions entirely through direct conditional dependencies, with edge magnitudes (0.260 and 0.158) larger than their corresponding mean absolute SHAP values (0.064 and 0.034). The remaining eight predictors had no direct edge to knee pain after regularization (Figure 5), indicating that their contributions to the model output were accounted for by their conditional dependencies with other variables in the network. This pattern of two variables with direct dependencies and eight contributing only through indirect pathways is consistent with the multidimensional structure described by the *ICF* framework.

### Sensitivity Analysis

Excluding EQ-5D Pain/Discomfort (n=20 variables; EPV=15.7) preserved the qualitative pattern of findings (Table S4 in Multimedia Appendix 1, Figures S1 and S2 in Multimedia Appendix 1). The pooled best AUC was 0.724 for SVM (*Δ*AUC =+0.001 vs main analysis; Figure S1 in Multimedia Appendix 1). The top SHAP feature shifted from EQ-5D Pain/Discomfort to EQ-5D Utility, but the next-ranked variables (house estate, walking physical activity, and BMI) remained the same (Figure S2 in Multimedia Appendix 1). House estate retained a direct conditional dependency with knee pain (partial correlation=0.20). The Spearman correlation between SHAP importance and network strength remained close to zero (*ρ*=−0.10; *P*=.78), and the CS-coefficient for strength was 0.44. The principal findings, including the modest discrimination performance, the predominance of indirect pathways from physical performance and mental health variables to knee pain, and the divergence between SHAP and network rankings, did not depend on inclusion of EQ-5D Pain/Discomfort.

## Discussion

### Principal Findings

This study examined self-reported knee pain in 852 community-dwelling older adults in Hong Kong using ML and partial-correlation network analysis. Based on the *ICF* model, knee pain in older age is associated with body function and structure, activity, participation, environmental, and personal factors [6,9,34]. These findings support this multidimensional view. Across the 21 predictors covering seven *ICF* domains, no single variable was dominant, and the model output reflected a distributed contribution from the health-related quality of life, environmental, physical performance, physical activity, mental health, and sleep domains. These results agree with the biopsychosocial view of knee pain in older adults [35], and with the projected rise of knee osteoarthritis to 595 million prevalent cases globally by 2050 [4,36]. Combining SHAP and network analysis allowed the assessment of which variables relate to knee pain and how those variables relate to one another.

The six classification algorithms showed similar discrimination, with pooled AUC ranging from 0.692 to 0.723. LR (AUC 0.721, 95% CI, 0.691–.751) was within 0.002 of RF and 0.001 of SVM, and showed the lowest Brier score (0.200) among the six algorithms. These results agree with prior reports that ML did not outperform LR in clinical prediction studies [37], and with recent recommendations that adequate sample size and prespecified variable selection, rather than algorithmic complexity, are the main determinants of stable AI-based prediction [38]. The discrimination level (best AUC 0.723) was comparable with prior ML models for knee pain or knee osteoarthritis in older adults, in which reported AUCs ranged from 0.70 to 0.80 [39-41]. With mild class imbalance (36.9% vs 63.1%), algorithmic resampling can degrade calibration without improving discrimination [42]; imbalance-aware metrics (AUC, *F*_1_-score, MCC, and Brier score) were therefore used [43]. The convergence of LR and ML performance suggests that, when EPV is 14.95 and the predictor set is conceptually prespecified, the benefit of more flexible algorithms over a regularized LR is small in cross-sectional knee pain classification.

SHAP-based importance and PDPs showed the model-implied association of each variable with the predicted probability of knee pain. The three highest-ranked features were EQ-5D Pain/Discomfort (mean |SHAP|=0.064), EQ-5D Utility (0.038), and house estate (0.034). The remaining features clustered at lower magnitudes (mean |SHAP| range, 0.013‐0.019) and included variables from the physical activity, sociodemographic, smartphone-based physical performance, mental health, and sleep domains. The SHAP-derived directions of association agreed with the baseline comparisons: lower walking-related physical activity and sit-to-stand repetitions, higher BMI and sitting time, lower mental health scores, and a higher count of sleep disorder categories were each associated with an increased predicted probability of knee pain. These patterns agree with prior reports linking reduced gait speed and lower-extremity capacity to musculoskeletal pain in older adults [44], and with the bidirectional association between sleep disturbance and chronic musculoskeletal pain [45]. Because PDPs show model-implied marginal associations rather than causal effects, the inflection points described below are reported as hypothesis-generating and require prospective validation. The predicted probability of knee pain rose sharply between EQ-5D Pain/Discomfort scores of 1 and 2 (0.31 to 0.43) and declined above an EQ-5D Utility of 0.85, while for the modifiable variables it declined steeply with walking physical activity up to 3000 MET·minutes/week, with sit-to-stand performance up to 15 repetitions, and rose above a BMI of 22 kg/m² and above a sitting time of 150 minutes/day. These patterns are consistent with prior reports linking sedentary behavior, low physical activity, higher BMI, and reduced lower-extremity capacity to musculoskeletal pain in older adults, but the exact values should not be treated as clinical cutoffs.

Network analysis showed how variables relate to one another after adjusting for the rest of the network. In the pooled 11-node network, only two predictors had direct edges with the knee pain node after EBICglasso regularization: EQ-5D Pain/Discomfort (partial correlation=0.260) and house estate (0.158). The remaining nine edges were among predictors. The Spearman correlation between SHAP importance and network strength centrality across the 10 feature nodes was ρ=0.018, indicating that the two analyses ranked variables differently and captured distinct facets of the multidimensional structure. Sit-to-stand showed this difference clearly. Although its marginal SHAP magnitude was modest (mean |SHAP|=0.0168; rank 7 of 21) and no direct edge to knee pain survived regularization, sit-to-stand showed the highest strength centrality (0.679). Sit-to-stand therefore acted as a central physical performance hub whose conditional dependencies extended across multiple *ICF* domains, including body structure and function (BMI, partial correlation = −0.146; age, −0.260), mental and cognitive function (mental health, +0.128), and activity (walking physical activity, +0.150). This pattern is consistent with the role of the sit-to-stand test as an integrative measure of lower-extremity power, balance, and global physical capacity in older adults [46,47]. Sit-to-stand therefore appears not as a direct correlate of knee pain, but as a multidimensional hub that links body function, body structure, activity, and mental health variables, which agrees with the biopsychosocial pathways described in the *ICF* model [15,48,49]. The direct edge between house estate and knee pain reflects rental estate residency as an indicator of lower socioeconomic position in Hong Kong, and is consistent with the well-documented inverse association between socioeconomic status and chronic pain [50], and with reports that public rental housing in Hong Kong is associated with poorer health-related behaviors and chronic disease outcomes [51]. Beyond reflecting socioeconomic position, the housing categories in this cohort were defined primarily by income level rather than by physical or care-related features, so the mechanisms underlying the association with knee pain remain uncertain and warrant further study. The direct edge between EQ-5D Pain/Discomfort and knee pain partly reflects conceptual overlap, as both capture self-reported pain; however, the two are distinct constructs (general pain or discomfort versus knee-specific pain), and a sensitivity analysis excluding EQ-5D Pain/Discomfort preserved the qualitative pattern of findings, indicating that our conclusions do not depend on this predictor.

Several implications for clinical practice and future research are noted. First, the multidimensional pattern across *ICF* domains supports multidisciplinary, biopsychosocial assessment for older adults with knee pain, rather than the use of isolated impairment-based measures [34,35]. Second, the central network position of sit-to-stand, despite its weak direct association with knee pain, indicates that this measure was conditionally connected to body structure, mental health, and activity domains within the observed cross-sectional network, in which sit-to-stand was simultaneously connected to BMI and age, mental health, and walking physical activity. Sit-to-stand performance was therefore associated with multiple *ICF* domains in this network, a pattern that would be missed by considering its marginal SHAP rank alone and that prospective studies should test directly. This relationship is consistent with reports linking sit-to-stand power to functional outcomes in advanced knee osteoarthritis [26], and with the general role of sit-to-stand as a functional vital sign in geriatric assessment [46]. Third, the smartphone-based digital health system used in this study, validated for walking speed (*r*=0.975), knee flexion (*r*=0.907‐0.923), and test-retest reliability (intraclass correlation coefficient=0.86‐0.94) [16], provides a feasible method for measuring *ICF* body function and activity domain variables in community settings. Because these inferences are hypothesis-generating, independent external validation in separate cohorts is a necessary next step before any clinical implementation, alongside prospective longitudinal studies.

This study had several limitations. First, the cross-sectional design did not allow causal inference; the direct and indirect pathways describe conditional dependencies and model-implied associations, not mechanistic effects. Second, knee pain was measured by a single self-report item covering the preceding 3 months and did not distinguish etiology (osteoarthritis, rheumatoid arthritis, meniscal injury, or referred pain), severity, or laterality; medical diagnoses and medication use for knee pain (such as oral or topical nonsteroidal anti-inflammatory drugs, duloxetine, or intra-articular injections) were not ascertained and could not be included in the modeling. These omissions limit the clinical interpretability of the outcome and may contribute to the modest discrimination observed. Third, participants were recruited from eleven Hong Kong Housing Society estates by convenience sampling and were predominantly female (77.5%); generalizability to other regions, ethnicities, sex distributions, and socioeconomic strata is therefore limited. Fourth, no external validation was performed; nested 5×5 cross-validation provided unbiased internal estimates but not external transportability. Fifth, strength centrality showed acceptable bootstrap stability (CS-coefficient=0.36), but closeness and betweenness were unstable; these two measures were therefore not interpreted further. Sixth, the interpretive tools have inherent limits: PDPs show model-implied marginal associations, not clinical thresholds; SHAP values are model-dependent and may split importance among correlated predictors; and centrality metrics, especially closeness and betweenness, can be unstable in modest cross-sectional networks, which is why strength was pre-specified as the primary measure. Finally, the smartphone-based assessment used a research-prototype application that is not commercially available, and broader translation will require additional regulatory and engineering work. Future prospective and longitudinal studies with larger and more diverse cohorts, severity-graded outcomes, and independent external validation are required to test the hypotheses generated by this work.

### Conclusions

This study combined ML and partial-correlation network analysis to examine self-reported knee pain in community-dwelling older adults within the *ICF* framework. The six classification algorithms showed pooled AUC values ranging from 0.692 to 0.723, indicating modest discrimination across the predictor set. Network analysis showed that only EQ-5D Pain/Discomfort and house estate had direct edges with knee pain, while sit-to-stand acted as a central hub linking body function, body structure, mental health, and activity variables. These cross-sectional, hypothesis-generating findings may inform multidisciplinary biopsychosocial assessment, but not causal or intervention conclusions, pending prospective external validation.

## Supplementary material

10.2196/92466 Multimedia Appendix 1 The full list of baseline assessment variables by ICF domain, missing data summary, hyperparameter grids, SHAP-based feature importance for all 21 predictors, the pooled partial correlation matrix, and results of the sensitivity analysis excluding the EQ-5D Pain/Discomfort dimension

## Abbreviations

AUC: area under the receiver operating characteristic curve
EBICglasso: extended Bayesian Information Criterion graphical LASSO
EPV: events per variable
EQ-5D: EuroQol 5-Dimension
*ICF*: *International Classification of Functioning, Disability and Health*
LASSO: Least Absolute Shrinkage and Selection Operator
LightGBM: Light Gradient Boosting Machine
LR: logistic regression
MCC: Matthews Correlation Coefficient
ML: machine learning
PDP: partial dependence plot
RF: random forest
SHAP: SHapley Additive exPlanations
SVM: support vector machine
XGBoost: Extreme Gradient Boosting
