# Supplementary material for: Smartphone-Based Physical Performance and Multidimensional Determinants of Self-Reported Knee Pain in Community-Dwelling Older Adults: Cross-Sectional Machine Learning and Network Analysis Study
Source: JMIR Mhealth Uhealth. 2026 Aug 3;14:e92466. doi: 10.2196/92466 (PMC13433902; doi:10.2196/92466)
Supplement: Supplementary file 1 [file mhealth-v14-e92466-s001.docx]

**Supplementary Materials**

**Contents**

**Supplementary Table S1.** Comprehensive baseline assessment variables organized by ICF domain

**Supplementary Table S2.** Missing data summary for the 21 retained predictor variables

**Supplementary Table S3.** Hyperparameter grids for nested cross-validation tuning

**Supplementary Table S4.** Sensitivity analysis excluding the EQ-5D Pain/Discomfort dimension

**Supplementary Table S5.** SHAP-based feature importance for all 21 predictor variables

**Supplementary Table S6.** Pooled partial correlation matrix for the 11-node network

**Supplementary Figure S1.** Sensitivity analysis for AUC comparison with and without EQ-5D Pain/Discomfort

**Supplementary Figure S2.** Sensitivity analysis for SHAP feature importance comparison

**Supplementary Table S1. Comprehensive baseline assessment variables organized by ICF domain**

The 38 variables collected at baseline were reduced to 21 candidate predictors prior to modeling using pre-specified, rule-based criteria (see Methods §2.4.1). Each variable is mapped to one of seven ICF-aligned domains with corresponding description and measurement instrument.

| **#** | **Variable** | **Display name** | **Type** | **Description** | **Instrument and Method** |
| --- | --- | --- | --- | --- | --- |
| ***Sociodemographic and anthropometric (3 retained variables)*** | | | | | |
| 1 | Age | Age | Continuous | Participant age in years | Structured interview |
| 2 | Sex | Sex | Categorical | Male / Female | Structured interview |
| 3 | Body mass index | BMI | Continuous | Weight / (Height)² in kg/m² | Standardized measurements using portable stadiometer and calibrated digital scale |
| ***Environmental (1 retained variable)*** | | | | | |
| 4 | Housing type | House estate | Categorical | Elderly housing vs rental estate (rental estates serve lower-income households; senior/elderly housing serves middle- to higher-income groups) | Recruitment record from Hong Kong Housing Society |
| ***Smartphone-based physical performance (4 retained variables)*** | | | | | |
| 5 | Walking speed | Walking speed | Continuous | Steady-state walking speed in m/s, computed from pelvis position over an 8-meter walkway | Vision-based smartphone application with computer vision pose estimation; Android device with single RGB camera mounted on a 0.9-meter stand placed 4 meters perpendicular to the midpoint of an 8-meter walkway; Python-based pose detection backend with MySQL database storage |
| 6 | Sit-to-stand | Sit-to-stand | Continuous | Number of complete sit-to-stand repetitions over 30 seconds, detected from pelvis vertical position and bilateral knee flexion angles | Same smartphone-based system as above |
| 7 | Gait knee flexion | Gait knee flexion | Continuous | Bilateral mean peak knee flexion angle during the early stance phase of gait, in degrees | Same smartphone-based system as above |
| 8 | Gait asymmetry | Gait asymmetry | Continuous | Absolute bilateral asymmetry of knee flexion angle (%) during gait | Same smartphone-based system as above |
| ***Physical activity (4 retained variables; from IPAQ short form)*** | | | | | |
| 9 | Sitting time | Sitting (min/day) | Continuous | Average sitting time per day in minutes | International Physical Activity Questionnaire (IPAQ) short form with sedentary behavior assessment |
| 10 | Walking PA | Walking PA | Continuous | Weekly MET-minutes of walking activity | IPAQ short form with standard MET-minute calculations |
| 11 | Moderate PA | Moderate PA | Continuous | Weekly MET-minutes of moderate-intensity activity | IPAQ short form with standard MET-minute calculations |
| 12 | Vigorous PA | Vigorous PA | Continuous | Weekly MET-minutes of vigorous-intensity activity | IPAQ short form with standard MET-minute calculations |
| ***Health-related quality of life (6 retained variables; EQ-5D-5L)*** | | | | | |
| 13 | EQ-5D Mobility | EQ-5D Mobility | Ordinal | Mobility dimension (1–5 scale; 1 = no problems, 5 = unable / extreme problems) | EQ-5D-5L questionnaire (Traditional Chinese version) with Hong Kong-specific value set |
| 14 | EQ-5D Self-care | EQ-5D Self-care | Ordinal | Self-care dimension (1–5 scale) | Same as above |
| 15 | EQ-5D Usual activities | EQ-5D Usual activities | Ordinal | Usual activities dimension (1–5 scale) | Same as above |
| 16 | EQ-5D Pain/Discomfort | EQ-5D Pain/Discomfort | Ordinal | Pain/Discomfort dimension (1–5 scale) | Same as above |
| 17 | EQ-5D Anxiety/Depression | EQ-5D Anxiety/Depression | Ordinal | Anxiety/Depression dimension (1–5 scale) | Same as above |
| 18 | EQ-5D Utility score | EQ-5D Utility | Continuous | Health utility score (−0.442 to 1.0) derived from the five EQ-5D dimensions using the Hong Kong value set | EQ-5D-5L value set for Hong Kong |
| ***Mental and cognitive (2 retained variables)*** | | | | | |
| 19 | Mental health (ReQoL) | Mental health | Continuous | ReQoL-10 total score (0–40), assessing recovering quality of life across mood, relationships, physical health, and daily functioning | Recovering Quality of Life 10-item questionnaire (ReQoL-10) with 5-point Likert scale |
| 20 | ERA-12 total | ERA total | Continuous | Total Expectations Regarding Aging score (12–48) | ERA-12 survey with 4-point Likert scale across three domains (cognitive function, physical health, mental health) |
| ***Sleep (1 retained variable)*** | | | | | |
| 21 | Sleep disorders count | Sleep disorders | Continuous | Total number of positive sleep disorder categories (0–6): insomnia, psychiatric, circadian rhythm, movement, parasomnia, sleep apnea | Sleep Disturbance Questionnaire (SDQ) with binary scoring across six diagnostic domains |

BMI, body mass index; ERA-12, 12-item Expectations Regarding Aging questionnaire; EQ-5D-5L, EuroQol 5-Dimension 5-Level; ICF, International Classification of Functioning, Disability and Health; IPAQ, International Physical Activity Questionnaire; MET, metabolic equivalent of task; PA, physical activity; ReQoL, Recovering Quality of Life; SDQ, Sleep Disturbance Questionnaire.

**Supplementary Table S2. Missing data summary for the 21 retained predictor variables**

Item-level missingness for each predictor in the analytic cohort (N = 852). Variables with no missing data are not shown. All missing values were addressed using multiple imputation by chained equations (m = 5; see Methods §2.4.2).

| **Variable** | **N missing** | **% missing** | **ICF domain** |
| --- | --- | --- | --- |
| Walking PA (MET-min/week) | 16 | 1.88 | Physical activity |
| ERA-12 total | 14 | 1.64 | Mental and cognitive |
| Mental health (ReQoL) | 13 | 1.53 | Mental and cognitive |
| Sleep disorders count | 13 | 1.53 | Sleep |
| Sitting time (min/day) | 11 | 1.29 | Physical activity |
| Moderate PA (MET-min/week) | 11 | 1.29 | Physical activity |
| EQ-5D Mobility | 10 | 1.17 | Health-related QoL |
| EQ-5D Self-care | 10 | 1.17 | Health-related QoL |
| EQ-5D Usual activities | 10 | 1.17 | Health-related QoL |
| EQ-5D Pain/Discomfort | 10 | 1.17 | Health-related QoL |
| EQ-5D Anxiety/Depression | 10 | 1.17 | Health-related QoL |
| EQ-5D Utility | 10 | 1.17 | Health-related QoL |
| Gait knee flexion | 9 | 1.06 | Smartphone PP |
| Gait asymmetry | 9 | 1.06 | Smartphone PP |
| Vigorous PA (MET-min/week) | 9 | 1.06 | Physical activity |
| Sit-to-stand | 4 | 0.47 | Smartphone PP |
| Walking speed | 1 | 0.12 | Smartphone PP |

Variables sorted by descending number of missing values. Maximum missingness 1.88%; median 1.17%. PP, physical performance.

**Supplementary Table S3. Hyperparameter grids for nested cross-validation tuning**

Hyperparameter grids searched within the inner 5-fold cross-validation of each algorithm. The combination maximizing inner-fold AUC was applied to the outer test fold; this procedure was repeated independently across 5 outer folds and 5 multiply-imputed datasets.

| **Algorithm** | **Hyperparameter grid** | **# combinations** |
| --- | --- | --- |
| **Logistic regression (LR)** | alpha (mixing): 0, 0.5, 1; lambda (regularization): 10⁻⁴ to 10¹ (8 values, log-spaced) | 24 |
| **k-Nearest Neighbors (kNN)** | kmax: 5, 11, 21, 31, 51; distance: 1, 2; kernel: rectangular, optimal, triangular | 30 |
| **Random Forest (RF)** | mtry: 2, 4, 6, 8; min.node.size: 1, 3, 5, 10; splitrule: gini | 16 |
| **Support Vector Machine (SVM, RBF)** | C: 0.01, 0.1, 1, 10, 100; sigma: 0.001, 0.01, 0.05, 0.1 | 20 |
| **XGBoost (XGB)** | nrounds: 200, 500; max_depth: 3, 5; eta: 0.05, 0.1; subsample: 0.8; colsample_bytree: 0.8 | 8 |
| **LightGBM** | num_leaves: 15, 31; learning_rate: 0.05, 0.1; nrounds: 200, 500; min_data_in_leaf: 10, 20 | 16 |

RBF, radial basis function. Hyperparameters not listed here used package defaults. Continuous predictors were centered and scaled within each cross-validation fold.

**Supplementary Table S4. Sensitivity analysis excluding the EQ-5D Pain/Discomfort dimension**

EQ-5D Pain/Discomfort shares conceptual content with the self-reported knee pain outcome. To assess robustness to this overlap, the full pipeline was repeated using only 20 predictor variables (EPV = 15.7). Side-by-side comparison of key outputs:

| **Outcome** | **Main analysis (n = 21)** | **Sensitivity (n = 20)** |
| --- | --- | --- |
| **EPV** | 14.95 | 15.70 |
| **Best algorithm (pooled AUC)** | RF | SVM |
| **Best AUC value** | 0.723 | 0.724 (ΔAUC = +0.001) |
| **Top SHAP feature** | EQ-5D Pain/Discomfort | EQ-5D Utility |
| **Top-3 SHAP features** | EQ-5D Pain/Discomfort; EQ-5D Utility; House estate | EQ-5D Utility; House estate; EQ-5D Anxiety/Depression |
| **Network: # direct edges to knee pain** | 2 | 1 |
| **Network: knee pain strength rank** | 5 of 11 | 7 of 11 |
| **Network: CS-coefficient (strength)** | 0.362 | 0.439 |
| **Spearman ρ (SHAP, Strength)** | 0.018 (P = .973) | −0.104 (P = .776) |
| **Spearman ρ (SHAP, Closeness)** | −0.033 (P = .948) | −0.100 (P = .950) |

EPV, events per variable. The qualitative pattern of findings was preserved: ML did not outperform LR; House estate retained a direct conditional dependency with knee pain; SHAP-centrality concordance remained near zero across both analyses, supporting the interpretation that the two paradigms capture distinct facets of the multidimensional structure.

**Supplementary Table S5. SHAP-based feature importance for all 21 predictor variables**

Mean absolute SHAP values, pooled across 5 multiply-imputed datasets, computed on the random forest model (best-performing caret-compatible algorithm). Higher values indicate greater contribution to the model output.

| **Rank** | **Variable** | **Mean \| SHAP \|** | **ICF domain** |
| --- | --- | --- | --- |
| 1 | EQ-5D Pain/Discomfort | 0.0641 | Health-related QoL |
| 2 | EQ-5D Utility | 0.0384 | Health-related QoL |
| 3 | House estate | 0.0344 | Environmental |
| 4 | Walking PA | 0.0191 | Physical activity |
| 5 | Body mass index | 0.0187 | Sociodemographic and anthropometric |
| 6 | Sitting time (min/day) | 0.0182 | Physical activity |
| 7 | Sit-to-stand | 0.0168 | Smartphone-based PP |
| 8 | Gait knee flexion | 0.0143 | Smartphone-based PP |
| 9 | Age | 0.0135 | Sociodemographic and anthropometric |
| 10 | Mental health (ReQoL) | 0.0133 | Mental and cognitive |
| 11 | Sleep disorders | 0.0132 | Sleep |
| 12 | Gait asymmetry | 0.0130 | Smartphone-based PP |
| 13 | Walking speed | 0.0128 | Smartphone-based PP |
| 14 | ERA-12 total | 0.0106 | Mental and cognitive |
| 15 | EQ-5D Mobility | 0.0097 | Health-related QoL |
| 16 | Vigorous PA | 0.0092 | Physical activity |
| 17 | Moderate PA | 0.0088 | Physical activity |
| 18 | Sex | 0.0073 | Sociodemographic and anthropometric |
| 19 | EQ-5D Anxiety/Depression | 0.0038 | Health-related QoL |
| 20 | EQ-5D Usual activities | 0.0025 | Health-related QoL |
| 21 | EQ-5D Self-care | 0.0007 | Health-related QoL |

SHAP values were computed using the fastshap package with 50 Monte Carlo permutation samples. PP, physical performance.

**Supplementary Table S6. Pooled partial correlation matrix for the 11-node network**

Partial correlations averaged across 5 multiply-imputed datasets, after EBICglasso regularization (gamma = 0.5) and edge thresholding. Off-diagonal values represent direct conditional dependencies between variable pairs after controlling for all other variables in the network. Zeros indicate edges that did not survive regularization.

|  | **EQ-5D PD** | **House** | **Walk PA** | **BMI** | **Sit** | **STS** | **Gait flex** | **Age** | **Mental** | **Sleep** | **Knee** |
| --- | --- | --- | --- | --- | --- | --- | --- | --- | --- | --- | --- |
| **EQ-5D Pain/Discomfort** | — | 0 | 0 | 0 | 0 | 0 | 0 | 0 | -0.17 | 0.24 | 0.26 |
| **House estate** | 0 | — | 0 | 0.21 | 0 | 0 | 0 | 0 | 0 | 0 | 0.16 |
| **Walking PA** | 0 | 0 | — | 0 | -0.05 | 0.15 | 0 | 0 | 0 | 0 | 0 |
| **BMI** | 0 | 0.21 | 0 | — | 0 | -0.15 | 0 | 0 | 0 | 0 | 0 |
| **Sitting (min/day)** | 0 | 0 | -0.05 | 0 | — | 0 | 0 | 0 | 0 | 0 | 0 |
| **Sit-to-stand** | 0 | 0 | 0.15 | -0.15 | 0 | — | 0 | -0.26 | 0.13 | 0 | 0 |
| **Gait knee flexion** | 0 | 0 | 0 | 0 | 0 | 0 | — | 0 | 0 | 0 | 0 |
| **Age** | 0 | 0 | 0 | 0 | 0 | -0.26 | 0 | — | 0 | 0 | 0 |
| **Mental health** | -0.17 | 0 | 0 | 0 | 0 | 0.13 | 0 | 0 | — | -0.23 | 0 |
| **Sleep disorders** | 0.24 | 0 | 0 | 0 | 0 | 0 | 0 | 0 | -0.23 | — | 0 |
| **Knee pain** | 0.26 | 0.16 | 0 | 0 | 0 | 0 | 0 | 0 | 0 | 0 | — |

Color coding: positive partial correlations in green; negative in red. Column headers (left to right): EQ-5D PD = EQ-5D Pain/Discomfort; House = House estate; Walk PA = Walking physical activity; Sit = Sitting (min/day); STS = Sit-to-stand; Gait flex = Gait knee flexion; Mental = Mental health; Sleep = Sleep disorders; Knee = Knee pain.

**Supplementary Figure S1. Sensitivity analysis for AUC comparison with and without EQ-5D Pain/Discomfort. (A) AUC comparison; (B) ΔAUC after excluding EQ-5D Pain/Discomfort.**

Side-by-side comparison of pooled performance between the main analysis (21 retained variables) and the sensitivity analysis excluding EQ-5D Pain/Discomfort (20 retained variables). The figure comprises two panels. AUC, area under the receiver operating characteristic curve; CI, confidence interval; EQ-5D PD, EQ-5D-5L Pain/Discomfort; kNN, k-nearest neighbors; LightGBM, light gradient boosting machine; LR, logistic regression with elastic-net penalty; RF, random forest; SVM, support vector machine with radial basis function kernel; XGB, extreme gradient boosting.

**Supplementary Figure S2. Sensitivity analysis for SHAP feature importance comparison.**

Removing EQ-5D Pain/Discomfort shifted the top-ranked feature from EQ-5D Pain/Discomfort to EQ-5D Utility, while the next-ranked variables (house estate, walking physical activity, body mass index) remained consistent between analyses. This pattern indicates that the contribution of non-pain predictors to the model output did not depend on the inclusion of the EQ-5D Pain/Discomfort dimension. BMI, body mass index; EQ-5D, EuroQol 5-Dimension 5-Level; PA, physical activity; ReQoL, Recovering Quality of Life; SHAP, SHapley Additive exPlanations.
